# Supplementary material for: Exploring the coupling coordination relationship and obstacle factors of rural revitalization, new-type urbanization, and digital economy in China
Source: PLoS One. 2025 Jan 16;20(1):e0313125. doi: 10.1371/journal.pone.0313125 (PMC11737860; doi:10.1371/journal.pone.0313125)
Supplement: Attachment — Submitted filename: PONE-D-24-22029.docx [file pone.0313125.s001.docx]

The manuscript entitled “Exploring the Coupled Coordination Relationship and Barriers of Rural Revitalization, New Urbanization and Digital Economy in China. (PONE-D-24-22029)” has investigated the rural revitalization (RR), new urbanization (NU) and digital economy (DE) systems, and investigates the spatial and temporal evolution characteristics and obstacle factors of the integrated systems in 31 provinces in China from 2011 to 2021 using the coupled coordination degree (CCD) model and obstacle degree (OD) model. However, there are still some formatting and logical problems in the article, and the following issues should be considered in the revision:

1. There are many problems with the use of English. For example, there are numerous grammatical and typographical errors scattered throughout the manuscript (e.g., composite is used inappropriately and should be replaced with comprehensive, “comprehensive development level” is repeated too often throughout the text and should be simplified or replaced appropriately, and in line 12 “The rural revitalization (RR), new type urbanization (NU), and digital economy (DE).”: There should be no comma between “new type” and “urbanization”, line 479 “the coupling coordination level transitionsfroms lightly unbalanced to barely balanced development, growing from 0.3696 in 2011 to 0.5232 in 2020, with a growth rate of 41.57%": in this sentence, ‘with a growth rate of 41.57%’ should be adjusted before the main sentence). There are many other similar low-level errors in this article, so I won't go into details. The author needs to polish the whole article carefully.

2. The literature review in the introduction is too general. It is recommended to further elaborate the relationship between the limitations of existing digital economy research and the marginal contribution of this paper.

3. The logical framework diagram in Fig.1 lacks specific explanations, for example, smart city construction does not seem to be mentioned in the paper.

4. Construction of the indicator system, Table1 of the Index indicator selection based on what?

5. In the “Result analysis” section, data tables or data sources should be given for line graphs, and data and plotting methods should be given for heat maps.

6. It is suggested to add a short summary at the beginning of each section, stating the main content and purpose of the section

7. In the Barrier Degree Analysis, examples using actual data are given to illustrate how the barrier degree is calculated and interpreted.

8. In the Conclusions section, the conclusions are too general and should be summarized with actual data.
