# Supplementary material for: Exploring the coupling coordination relationship and obstacle factors of rural revitalization, new-type urbanization, and digital economy in China
Source: PLoS One. 2025 Jan 16;20(1):e0313125. doi: 10.1371/journal.pone.0313125 (PMC11737860; doi:10.1371/journal.pone.0313125)
Supplement: Attachment — Submitted filename: Response to Reviewers.docx [file pone.0313125.s002.docx]

**Response to Reviewer Comments**

Dear reviewers,

Thank you very much for your careful review and detailed comments on our paper. The constructive suggestions helped us immensely in our revision. We have carefully considered these comments and answered all the questions one by one. We revised the manuscript accordingly. The main revised part is highlighted in red in the manuscript.

Thank you and best regards!

Yours Sincerely,

Authors

**Reviewer #1**

● *1- -* (1) There are still some problems in the article, for example, the introduction needs to introduce the research significance of the article from a global perspective.
Response: Thank you for your comments. The introductory part of the article adds the significance of exploring the coupling relationship between the three systems of rural revitalization-new urbanization-digital economy from a global perspective

**Modified Introduction:**

Inequality is a global concern of the United Nations Sustainable Development Goals, and the digital economy (DE) is helping to reduce a country’s income gap on a global scale 1. With the arrival of the digital economy, the number of global Internet users has reached 5.373 billion. The digital economy breaks the limitations of traditional modes, weakens the asymmetry of information access, and becomes an efficient driving force for economic efficiency and quality improvement 2. The United Nations introduced the Sustainable Development Goals (SDGs), which, in order to accelerate progress and achievements, specifically emphasize the importance of the countryside and promote rural revitalization (RR) as a critical strategic objective 3,4. The ultimate goal of the rural revitalization strategy is common prosperity, which solves the problem of unbalanced development between urban and rural areas. New-type urbanization (NU), as an exogenous power of the countryside, promotes the flow of talents, science and technology, capital, information, and other factors to the countryside, stimulates the endogenous power of the countryside, and realizes the revitalization of the countryside. RR is the foundation and premise of new urbanization, which brings new space carriers and demand power for the development of DE. At the same time, DE provides new kinetic energy, such as information and resources for the development of rural revitalization. In the process of promoting rural revitalization, accelerating new urbanization and developing digital economy, exploring the coupled and coordinated relationship, spatial and temporal evolution pattern, and the obstacle factors of the coordinated development in the RR-NU-DE system will help to accelerate the realization of a series of goals, such as China’s economic transformation, digitalization, urban-rural integration, and rural revitalization, and at the same time refer to the digital economy and urban-rural integration in the world. From a global perspective, many countries are currently facing unbalanced development between urban and rural areas, aging populations, technological backwardness and other dilemmas, and exploring the coupling relationship between rural revitalization, new urbanization and the digital economy, as well as the main obstacles, can effectively reduce the gap between urban and rural areas, improve the overall competitiveness of the country, and promote the flow of resources between regions, thereby promoting the balanced development of all countries globally and realizing the optimization of the allocation of resources globally.

● 2- - (2) The discussion part of the paper needs to be further deepened. What is the difference between the conclusions of this paper and those of existing research? It is necessary to analyze the reasons for the difference.

**Response:** Thank you for your comments. The article has been refined with a conclusion section that highlights the importance of this study by contrasting it with existing findings.

**Modified Discussions:**

According to the indicator system and analysis results of rural revitalization, new-type urbanization, and digital economy, the evolution of the coupling coordination degree level of the 31 provinces in the country is closely related to the rural revitalization, new-type urbanization, and digital economy of each province. The digital economy and urban-rural relations show a more coordinated trend over time 77. The digital economy has become the engine of rural revitalization development step by step, and the degree of coupling and coordination between the digital economy and rural revitalization is positively correlated with noticeable regional differences 7,83,84. China’s new-type urbanization and rural revitalization strategies have shifted from traditional territorial expansion to high-quality integration, with the degree of coupling and coordination between the two increasing and the quality of life in urban and rural areas consistent 85,86. These studies provide a theoretical basis for the coupled coordination degree relationship of rural revitalization, new-type urbanization, and digital economy, and when focusing on urban-rural development and digital economy development, policymakers and stakeholders must recognize the interactions between the three. As soon as possible, these studies have led to a deeper understanding of the relationship between urban and rural development and the digital economy. Still, the systemic integrity of the research has been neglected 46. The current study focuses on exploring the relationship between the two two systems. Related research is mostly theoretical mechanism research, a few literature will be coupled coordination degree model applied to the relationship between the two, not the organic combination of these three, to explore the synergistic development relationship; in addition, the relevant research has not explored the obstacle factors of the three coupling, through the exploration of the obstacle factors, which can help to provide a precise basis for decision-making for the coordinated development of the digital economy, the new type of urbanization and the revitalization of the countryside. Still, the digital economy has become an essential engine of global economic change and a driving force for China’s high-quality economic development. At the same time, rural revitalization and new-type urbanization are fundamental initiatives to solve the three rural issues 2,87-89, so the coupled and coordinated relationship between the three is the focus of our inquiry. In this study, the relationship between rural revitalization, new-type urbanization, and digital economy is examined in depth from multiple perspectives, including the comprehensive development level, the spatio-temporal evolution of the coupling coordination degree, and the obstacle factors, with the aim of comprehensively and systematically evaluating and analyzing the coupling relationship between the digital economy and urban-rural development. Explore the obstacle factors for coupling the three, assist in high-quality economic development, green and sustainable development, and narrowing the gap between urban and rural areas in new urbanization and rural revitalization, and find opportunities for the innovative development of the digital economy.

● *3- -* (3) The language of the article still needs polishing.

**Response:** Thank you for your comments. We have carefully checked the language of the full manuscript. And we also have revised the abstract. According to your advice, this manuscript was edited for proper English language, grammar, punctuation, spelling, and overall style by one or more highly qualified native English speakers.

● *4- -* (4) The vast majority of references are articles by Chinese scholars, and the small number of articles by scholars from other countries indicates that scholars from other countries are not interested in the topic?

**Response:** Thank you for your insightful comments regarding the references cited in our manuscript. We appreciate your observation about the predominance of articles by Chinese scholars and the implications it may have on the perceived interest of international researchers in this topic. In response, we will expand our literature review to include a broader range of studies from scholars outside of China. This will provide a more comprehensive overview of global perspectives on the subject and demonstrate the relevance and importance of our research within the international academic community. We believe that incorporating a wider array of references will strengthen our work and reflect the global significance of our study. Thank you once again for your valuable feedback.
